# Supplementary material for: Blood-Flow Restriction Resistance Exercise for Older Adults with Knee Osteoarthritis: A Pilot Randomized Clinical Trial
Source: J Clin Med. 2019 Feb 21;8(2):265. doi: 10.3390/jcm8020265 (PMC6406824; doi:10.3390/jcm8020265)
Supplement: Supplementary file 1 [file jcm-08-00265-s001.zip › Supplementary Table.pdf]

Supplementary Table

Table S1. Clinical chemistry and hematology safety outcomes

	BFR	MIRT	Mean Difference
Urea Nitrogen (BUN), mg/dL	−2.02 (−3.92, −0.12)	0.21 (−1.79, 2.21)	−2.23 (−5.02, 0.55)
Sodium, mmol/L	−0.06 (−1.30, 1.17)	−1.99 (−3.29, −0.69)	1.93 (0.10, 3.75)
Creatinine, mg/dL	−0.02 (−0.08, 0.04)	0.05 (−0.01, 0.12)	−0.08 (−0.16, 0.01)
Potassium, mmol/L	−0.18 (−0.37, 0.02)	0.04 (−0.17, 0.25)	−0.21 (−0.50, 0.08)
Chloride, mmol/L	−0.33 (−1.48, 0.82)	−1.24 (−2.43, −0.06)	0.91 (−0.78, 2.60)
Carbon Dioxide, mmol/L	0.06 (−1.68, 1.80)	−0.08 (−1.93, 1.77)	0.14 (−2.43, 2.71)
Calcium, mg/dL	−0.09 (−0.25, 0.06)	−0.07 (−0.23, 0.09)	−0.02 (−0.26, 0.21)
Total Protein, g/dL	−0.14 (−0.34, 0.06)	−0.08 (−0.30, 0.13)	−0.06 (−0.36, 0.25)
Albumin, g/ml	0.04 (−0.09, 0.17)	0.00 (−0.13, 0.14)	0.04 (−0.15, 0.23)
Globulin, g/dL	−0.15 (−0.28, −0.02)	−0.13 (−0.27, 0.01)	−0.02 (−0.22, 0.18)
Total Bilirubin, mg/dL	0.04 (−0.08, 0.15)	−0.03 (−0.15, 0.09)	0.06 (−0.10, 0.23)
Alkaline Phosphate, U/L	−0.33 (−6.63, 5.67)	1.80 (−5.14, 8.74)	−2.13 (−11.75, 7.49)
Red Blood Cell Count, cell/L	−0.07 (−0.18, 0.04)	0.00 (−0.11, 0.11)	−0.07 (−0.23, 0.09)
White Blood Cell Count, cells/L	−0.42 (−0.85, 0.02)	0.37 (−0.10, 0.85)	−0.79 (−2.44, −0.13)
Hematocrit, L/L	0.29 (−1.78, 2.37)	−2.70 (−4.82, −0.58)	3.00 (−0.06, 6.05)
Platelet Count, cells/L	−17.02 (−33.37, −0.66)	3.56 (−13.66, 20.79)	−20.58 (−44.61, 3.44)

Clinical chemistry and hematology safety outcomes were evaluated from baseline to week 12. Values are reported as the estimate mean difference and 95% CI. Abbreviations: BFR—blood-flow restriction, MIRT—moderate-intensity resistance training.
