# Supplementary material for: Blood-Flow Restriction Resistance Exercise for Older Adults with Knee Osteoarthritis: A Pilot Randomized Clinical Trial
Source: J Clin Med. 2019 Feb 21;8(2):265. doi: 10.3390/jcm8020265 (PMC6406824; doi:10.3390/jcm8020265)
Supplement: Supplementary file 1 [file jcm-08-00265-s001.zip › Supplementary Figures.pdf]

SUPPLEMENTARY FIGURE LEGENDS

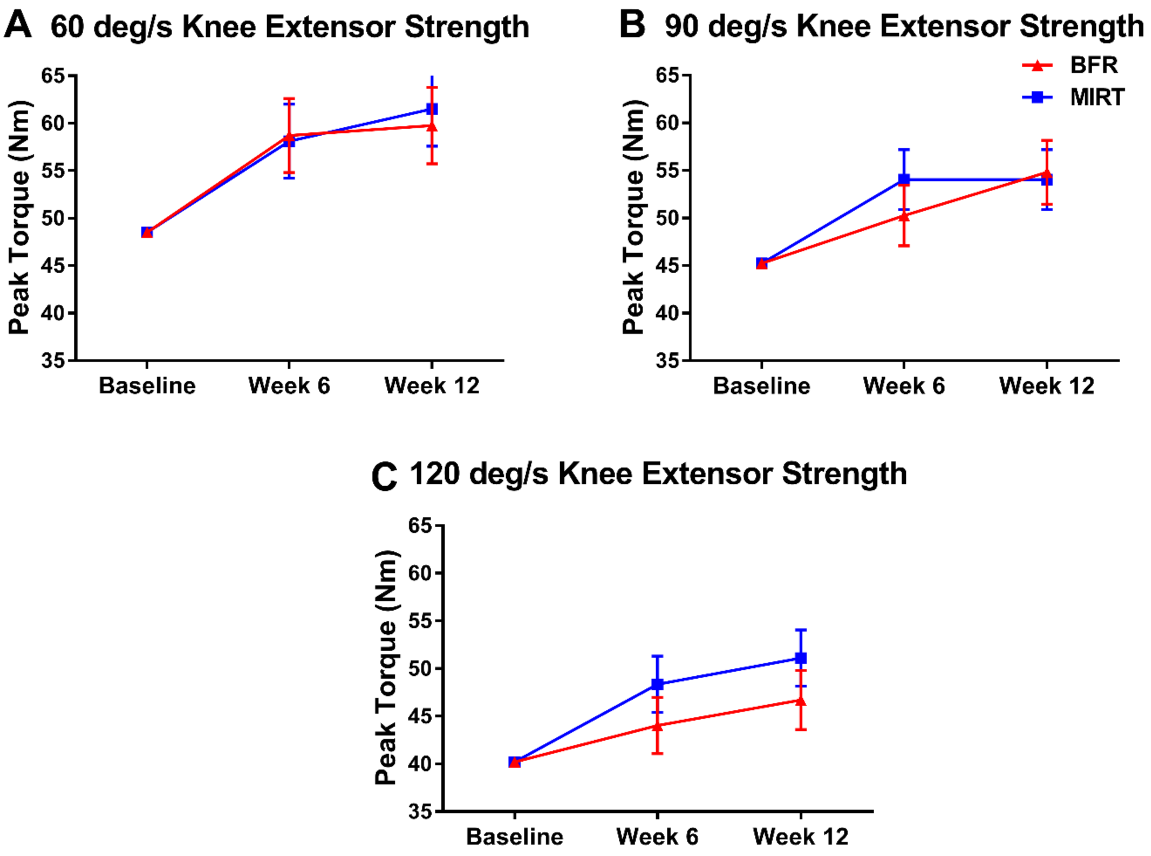

Figure S1 A, B, C. Change in knee extensor peak torque (Nm) by group across three speeds of movement: 60°/sec (A), 90°/sec (B), and 120°/sec (C) from baseline to week 12. Values indicate estimated marginal mean ± SEM. Abbreviations: BFR - blood-flow restriction, MIRT - moderate-intensity resistance training.

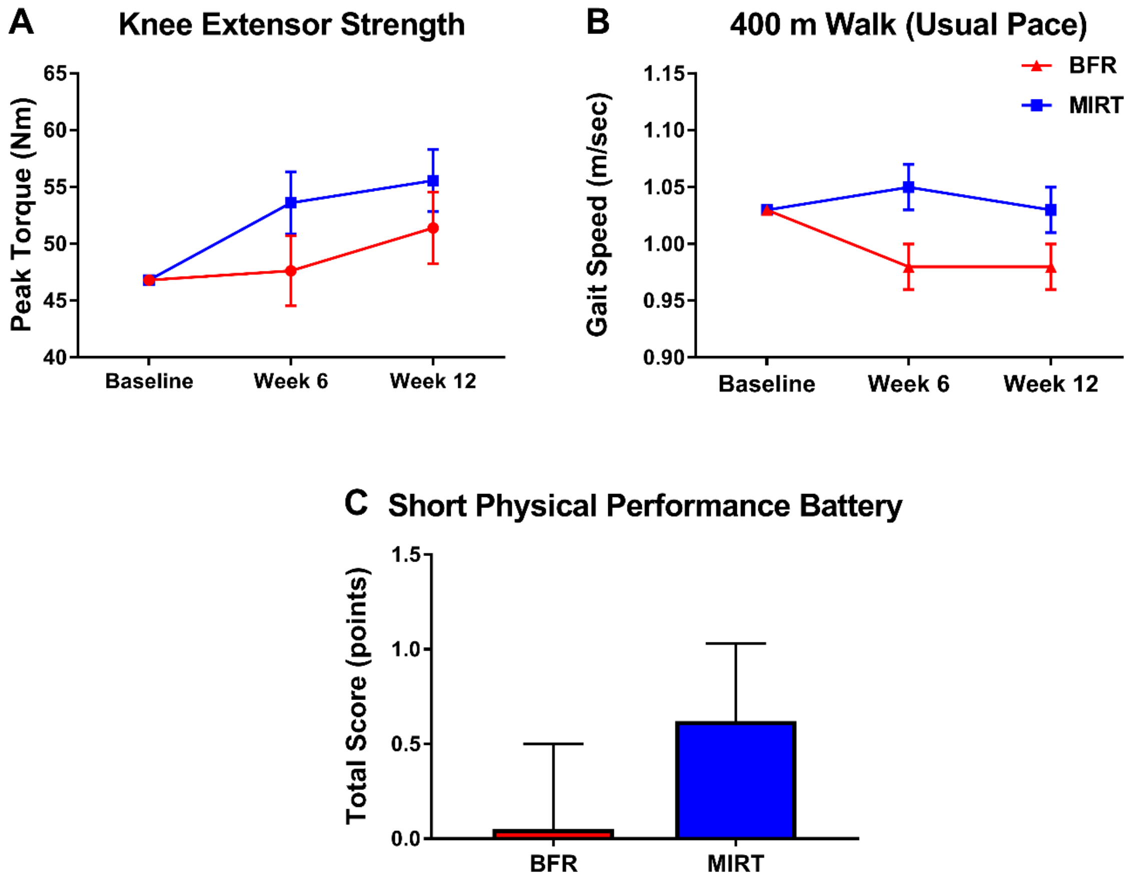

Figure S2 A, B, C. Efficacy analysis of objective measures of physical function from baseline to week 12 were evaluated by mean composite unilateral knee extensor peak torque (Nm) across three speeds of movement (60, 90, 120 °/sec) (A), 400 m walk usual pace gait speed (B), and Short Physical Performance Battery (C). Efficacy analysis was conducted among participants with ≥ 80% exercise attendance and attended baseline and week 12 study visits. Values indicate estimated marginal mean ± SEM. Abbreviations: BFR - blood-flow restriction, MIRT - moderate-intensity resistance training.

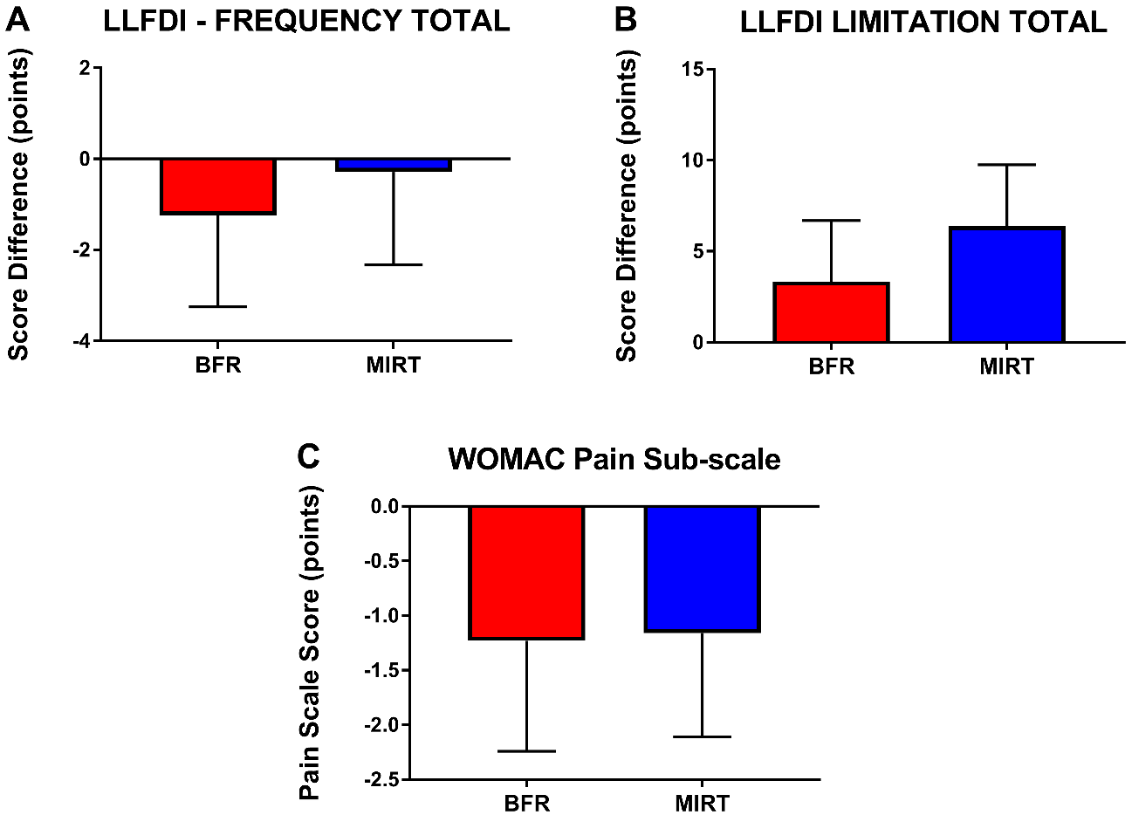

Figure S3 A, B, C. Efficacy analysis of subjective measures of pain and function were evaluated from baseline to week 12 via calculated group mean difference for Late Life Functional and Disability Instrument (LLFDI) Total Disability Frequency (A), Total Disability Limitation (B) where a higher reported score represents better performance, and Western Ontario & McMaster Universities Osteoarthritis Index (WOMAC) pain sub-scale (C). Efficacy analysis was conducted among participants with ≥ 80% exercise attendance and attended baseline and week 12 study visits. Values indicate estimated marginal mean ± SEM. Abbreviations: BFR - blood-flow restriction, MIRT - moderate-intensity resistance training.

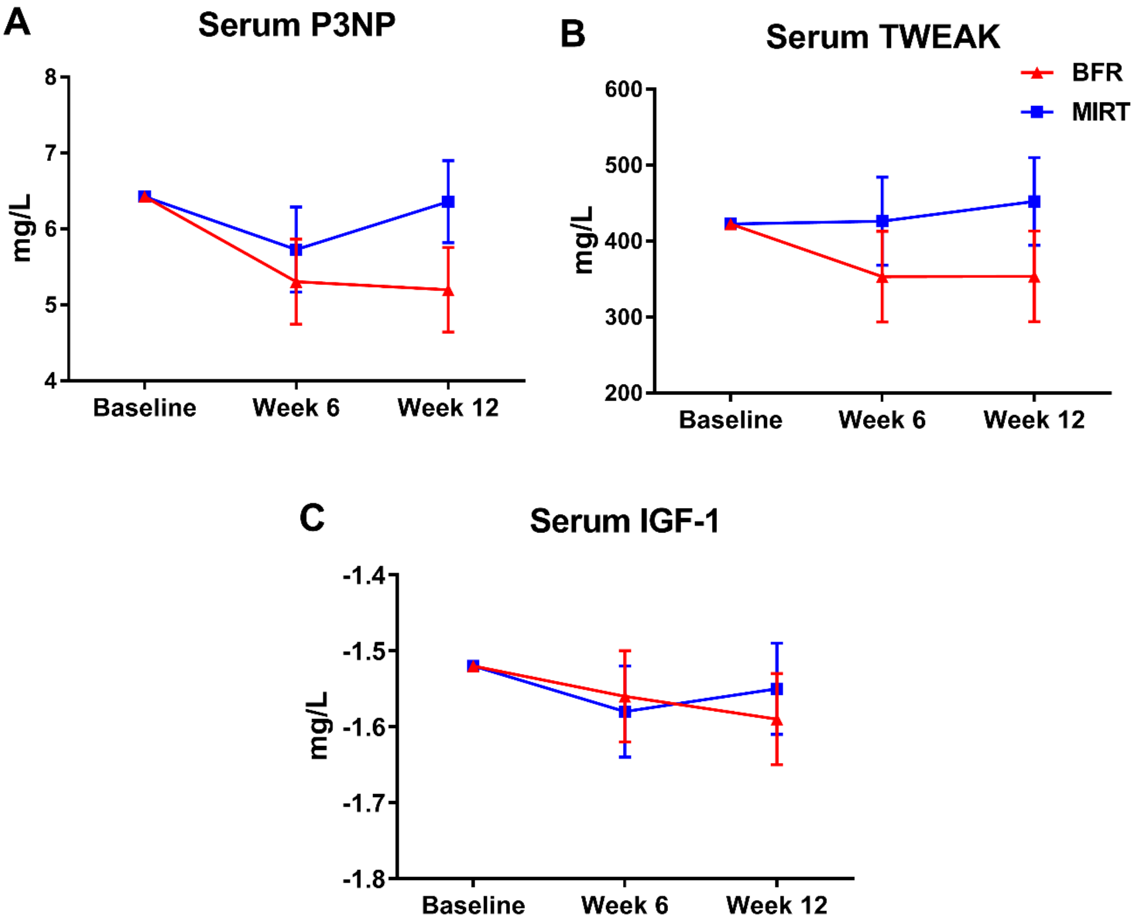

Figure 4 A, B, C. Efficacy analysis of serum biomarkers of myogenic activity and collagen turnover including N-terminal peptide of procollagen type III (P3NP) (A), tumor necrosis-like weak inducer of apoptosis (TWEAK) (B), and insulin-like growth factor (IGF-1) (C). Efficacy analysis was conducted among participants with ≥ 80% exercise attendance and attended baseline and week 12 study visits. Values indicate estimated marginal mean ± SEM. Abbreviations: BFR - blood-flow restriction, MIRT - moderate-intensity resistance training.
